# Case series of Rapidly growing Mycobacterial Post-operative surgical site infection in kidney transplant recipients

**DOI:** 10.1016/j.idcr.2022.e01640

**Published:** 2022-11-03

**Authors:** Muna Al Masalmani, Samar Mahmoud A. Hashim, Ajithkumar Ittaman, Sulieman S. Abu Jarir, Mohammed Abukhattab, Hussam Al Soub, Zubaida Al Suwaidi, Riyadh Fadhil, Omar Ali

**Affiliations:** a Infectious Diseases, Hamad Medical Corporation, Qatar; b Communicable Diseases Center, Qatar; c Urology Hamad Medical Corporation, Qatar; d Clinical Laboratory, Hamad Medical Corporation, Qatar

**Keywords:** Case report, NTM, MOTT, Kidney transplant, Post-operative surgical site infection

## Abstract

**Background:**

Case series of Rapidly growing Mycobacterial Post-operative wound infection with Mycobacterium Abscessus and/or Mycobacterium Chelone in 4 cases of kidney transplant adult recipient who presented within 6 months of transplant*.*

**Case presentations:**

We report 4 cases of Renal transplant recipients with post-surgical site infection with NTM-69-year-old with post-surgical wound infection with microbiologically proven Mycobacterium Abscessus who discontinued treatment and further presented with intra-abdominal abscess. Next case was 61 years male presented with nodular swellings at surgical site with US findings of intra-abdominal muscle abscess was tested culture positive for Mycobacterium Chelonae and Abscessus.Third case was 34 years male presented with surgical wound infection which was positive for AFB by ZN stain. Lastly,46 years old male patient known hypertensive and E.S.R.D, had culture proven Mycobacterium Abscessus surgical wound infection. All the four cases had their renal transplant at Philippines at different centres.

**Conclusions:**

Nontuberculous mycobacteria infection is important cause of morbidity in kidney transplant recipient and high index of suspicion with early diagnosis and treatment is crucial for successful outcome.

## Introduction

Diseases caused by non-tuberculous mycobacteria (NTM) have significance i in the current public health arena. Steep increases in the worldwide incidence and prevalence of these diseases are linked with the increasing numbers of patients with pulmonary *Mycobacterium avium* complex (MAC) disease in many countries. Currently, NTM consist of more than 150 species and they are globally ubiquitous in both natural and man-made environments. It is believed that NTM are generally acquired from the environment *via* ingestion, inhalation, and dermal contact, which results in lymphadenitis, pulmonary and disseminated infections, and skin and soft tissue infections. Water, soil, and dust have been reported to be MAC niches. Environmental MAC tends to gather in households, and in these niches, household tap water, bathrooms, potting soil, and garden soil are infection source. Although on immunosuppression including chronic steroids, the incidence of wound infections, is low in kidney recipients [1] and incidence is similar to other surgeries. However, in kidney recipients, use of MMF (vs. azathioprine) is an additional risk factor-one that potentially could be altered, especially in high-risk recipients.

Incidence estimates for all NTM diseases are (0.02–0.38 %) in renal,(0.24–2.8 %) in heart, (0.46–2.3 %) in lung and 0.1 % in liver transplant recipients [1]. Sites of NTM infection in renal transplant recipients are as follow: disseminated infection was most common (40.0 %), followed by cutaneous (32.2 %), lung (9.6 %) musculoskeletal (8.7 %), allograft (2.6 %), lymph node (2.6 %), gastrointestinal (1.7 %), for cardiovascular, reproductive and urinary system each (0.9 %) [2].

## Case reports

### Case: A

69 years old Qatari gentleman, Kidney transplant recipient from a live donor in 2007 in Philippines,with past history of diabetes mellitus type 2 and Hypertension presented to our institute outpatient clinic about 4 months after transplant with complaints of purulent discharge from surgical site infection for 1 week. Post-transplant period till then was uneventful and maintained on Prednisolone, Tacrolimus, and mycophenolate mofetil. He was afebrile and with stable vital signs. Physical examination revealed post-surgical wound infection with purulent discharge.

Ultrasonography abdomen showed no fluid collection in neither anterior abdominal wall or intra abdomen. Acid fast bacilli (A.F.B) was demonstrated by Ziehl-Neelsen (Z.N) stain in the pus swab specimen collected from the wound. Culture grew Mycobacterium abscessus sensitive to amikacin, clarithromycin. Intermediate to ciprofloxacin, cefoxitin, linezolid. Resistant to doxycycline. But patient was lost to follow up for 7 months.

Then he came with small abscess along the wound scar. This time, the Ultrasound showed subcutaneous and intramuscular pus collection in the right iliac fossa at the site of incision approximately measuring 3.2 × 2.8 centimetres. According to the sensitivity, he was started on intravenous amikacin, oral clarithromycin, and oral ciprofloxacin. Then after 2 weeks, amikacin was stopped, because of increase in creatinine Clarithromycin and ciprofloxacin were continued for another 1 month. his wound was getting better with less pus discharge.

Follow up after 1 year showed was that wound healed and no pus collection in ultrasonography of abdomen with normal kidney function.

### Case: B

A 61 years old gentleman kidney transplant recipient from a live donor in Philippines in 2007 with past history of diabetes mellitus and hypertension presented after three months after transplant with nodules over post-surgical site infection for 2 months. He had no other symptoms. He was maintained on mycophenolate mofetil, tacrolimus and prednisolone. Ultrasound abdomen showed large heterogenous intramuscular abdominal mass about 7.8 × 3 cm with focal nodular mass measuring 3 × 2.9 cm with rim of calcification. Aspiration from this fluid was done and ZN stain was positive for A.F.B.

Empirically patient was started on oral clarithromycin and ciprofloxacin. Pus Culture was positive for mycobacterium chelonae and abscessus and sensitivity showed, sensitive to amikacin, clarithromycin, and linezolid, intermediate to ciprofloxacin and cefoxitin and resistant to doxycycline. So, ciprofloxacin was stopped, and clarithromycin was continued for 6 months. He was followed up regularly and wound became clean and kidney function remained stable (Picture 1, Picture 2, Picture 3).

**Picture 1:**
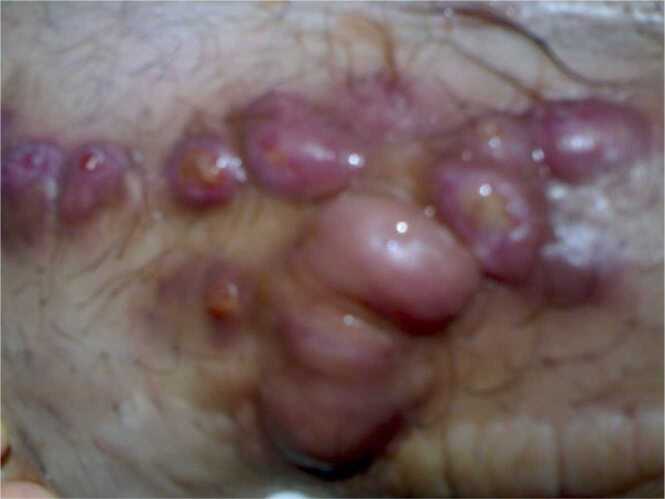
Nodular skin lesions at presentation.

**Picture 2:**
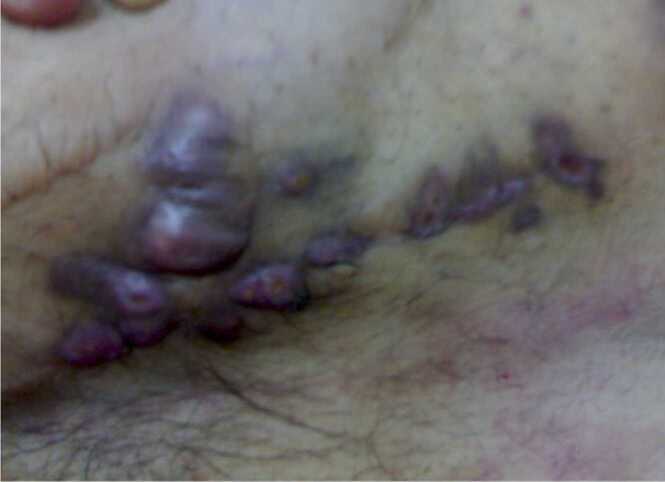
Regression of lesions during treatment.

**Picture 3:**
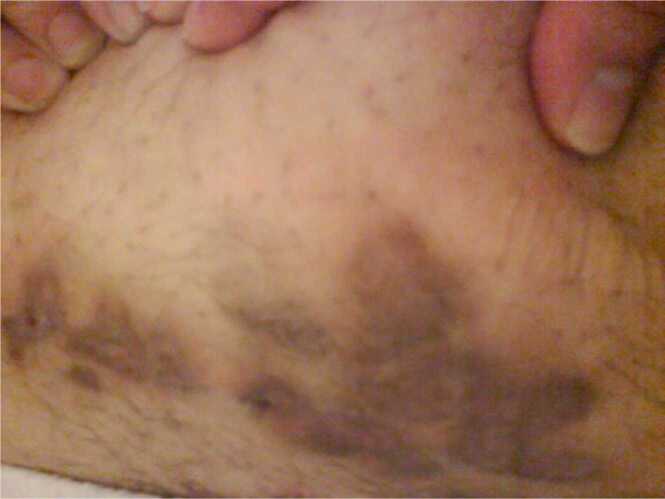
Healed lesion after complete therapy at 6 months.

### Case: C

34 years old gentleman known to have diabetes mellitus type 2, hypertension, status post kidney transplant in Philippines from live unrelated donor. His post-operative course was complicated. He had twice exploration 4th day post-surgery, for ureter leak which was repaired then at two weeks for urinary bladder leak which was also repaired. Three weeks post operatively he started to have purulent pus discharge from the post-surgical site infection, so there he had wound debridement. Patient arrived at our institute one month after surgery. He was afebrile but there was pus discharge from wound. He was maintained on prednisolone, mycophenolate mofetil and tacrolimus. Ultrasound abdomen: showed no collection around transplanted kidney, neither in abdomen. Pus discharge was positive for AFB by ZN stain, pending culture patient was started empirically on clarithromycin, moxifloxacin, linezolid. His wound culture grew E. coli and enterococcus faecalis and he had bacteraemia with E. coli. So, received IV antibiotics for total 14 days (intravenous piperacillin/tazobactam then intravenous amoxicillin/clavulanic acid).

Mycobacterium failed to grow from wound culture. Moxifloxacin and linezolid were stopped, and patient was continued on clarithromycin PO for 4 months. Upon follow up his wound healed with no more discharge.

### Case: D

46 years old gentleman patient known hypertensive and E.S.R.D, had kidney transplant from live unrelated donor in Philippines. Post-operatively patients had renal failure and he was back on haemodialysis. He was attended in our Transplant Infectious disease clinic at our institute three weeks post-transplant with pus discharge from surgical site infection. AFB was positive from wound discharge, so he was started empirically on moxifloxacin/rifampicin and clarithromycin. Culture grew mycobacterium chelone, sensitive to amikacin, clarithromycin. Intermediate to cefoxitin. Resistant to doxycycline, ciprofloxacin, and linezolid. Graft biopsy showed renal cortical necrosis, so he had graft nephrectomy after four months of transplant.

He had cytomegalovirus infection treated with intravenous ganciclovir, patient was maintained on oral clarithromycin and his wound healed.

He was maintained on clarithromycin as secondary prophylaxis till he had second kidney transplant, from deceased donor in China. Clarithromycin was continued for one year after second renal transplant surgery. There was no recurrence of NTM infection and renal function was stable.

## Discussion

Renal transplant recipients are at risk of developing NTM infections. The cell-mediated immune response, which is crucial in combating these infections, is impaired because of immunosuppression to prevent transplant rejection. Calcineurin inhibitor prevents the transcription of interleukin-2 (IL2) in the activated T cells, causing them to have reduced proliferation at the site of infection [3]. Mycobacterium chelonae, and Mycobacterium abscessus are environmental mycobacteria that can cause chronic infections of the skin, soft tissues, and lungs. These organisms are characterized by rapid growth on standard media and by lack of pigmentation [4]. in Solid organ transplant recipients,most common presentation of NTM infection is cutaneous followed disseminated disease [5]. In contrast to previous studies that mentioned, in general NTM infections are considered as late post-transplant infectious complication with mean of 48 months after transplantation [6].

In our case series the presentation of patients with NTM wound infection was early ranged from three weeks post-transplant in Case C to three months post-transplant in Case A. This finding can be explained as those infections have their origin during the surgical procedure. The presentation of surgical site infections due to NTM can be described in four stages [6].

**Table:** 

Stage	Clinical features
Stage 1	Tender nodules at surgical site after four weeks of the procedure.
Stage 2	The nodules get bigger in size and more tender and eventually form a sinus
Stage 3	There is a reduction of pain with necroses of overlying skin
Stage 4	The area develops into a chronic sinus
Stage 5	The area darkens with necrosed skin

In our case series, patients presented very early even before nodules formed except case 2 who presented with nodules at site of surgery.Early diagnosis of NTM infection in transplant recipients is critical because delays in diagnosis can lead to additional morbidity and possible mortality [5]. Clinical suspicion was high in these cases and so the samples were sent for gram stain culture and also for Ziehl-Neelsen (Z.N) stain.

To make a firm diagnosis of NTM disease, culture of representative clinical specimens and histological examination of tissue biopsy specimens are generally necessary. Culture in the gold standard for diagnosis of NTM disease. Multi department approach with efficient and prompt communication between Microbiologists, Clinicians and Infectious diseases specialist is necessary for early detection increase the sensitivity of culture and laboratory diagnosis of NTM disease [7].

Although the ZN method has relatively low sensitivity of detecting NTM in pus aspirates from wound infections [8],

All the four cases were positive by ZN. Culture of mycobacterium chelonae/abscessus was obtained in three cases except in Case C as it may be inhibited by bacterial growth. Antibiogram showed sensitivity to amikacin and clarithromycin. All the 4 cases were given clarithromycin. Although all cases were sensitive to amikacin, it was used for 2 weeks in Case A only because of the concern of nephrotoxicity. The antibiotics resistance is a major concern for NTM infections treatment and thus monotherapy is never recommended. Single-drug therapy may play a role only in special situations such as skin infection by M. chelonae and treated with clarithromycin^!^ [2].

Our reported cases showed favourable clinical response, despite treatment with monotherapy. Allograft function was preserved except in Case D as the patient had renal cortical necrosis which lead to graft nephrectomy. He had second kidney transplant and the new graft was functioning well with no recurrence of infection with regular clarithromycin secondary prophylaxis which was continued for 1 year after second graft.

All the four cases had their Renal transplant in Philippines, but at different centres over different time period.

Nontuberculous mycobacteria infection is important cause of morbidity in kidney transplant recipient and with high index of suspicion with multiteam approach is necessary for early detection and management.

## CRediT authorship contribution statement

**Muna Al Masalmani:** Main Author. **Samar Mahmoud A. Hashim:** Co-author, Writing – original draft, Writing – review & editing. **Ajithkumar Ittaman:** Co-author, Writing – original draft, Writing – review & editing. **Sulieman S. Abu Jarir:** Co-author. **Mohammed Abukhattab:** Co-author. **Hussam Al Soub:** Co-author. **ZubaidaAl Suwaidi:** co-author. **Riyadh Fadhil:** Co-author. **Omar Ali:** Co-author.

## Ethical approval

IRB, Hamad Medical Corporation.

## Consent

Yes; we have no conflict of interest to disclose consent.

## Funding

No funding for the study

## Funding for Publication

Qatar National Library funds the publication.

## Conflict of interest

Authors have no conflict of interest to disclose.
